# Supplementary material for: Ultra-sensitive and rapid detection of nucleic acids and microorganisms in body fluids using single-molecule tethering
Source: Nat Commun. 2020 Sep 22;11:4774. doi: 10.1038/s41467-020-18574-7 (PMC7508858; doi:10.1038/s41467-020-18574-7)
Supplement: Supplementary file 1 — Supplementary Information [file 41467_2020_18574_MOESM1_ESM.pdf]

**Ultra-sensitive and rapid detection of nucleic acids and microorganisms in body fluids using single molecule tethering**

Cheng *et al.*

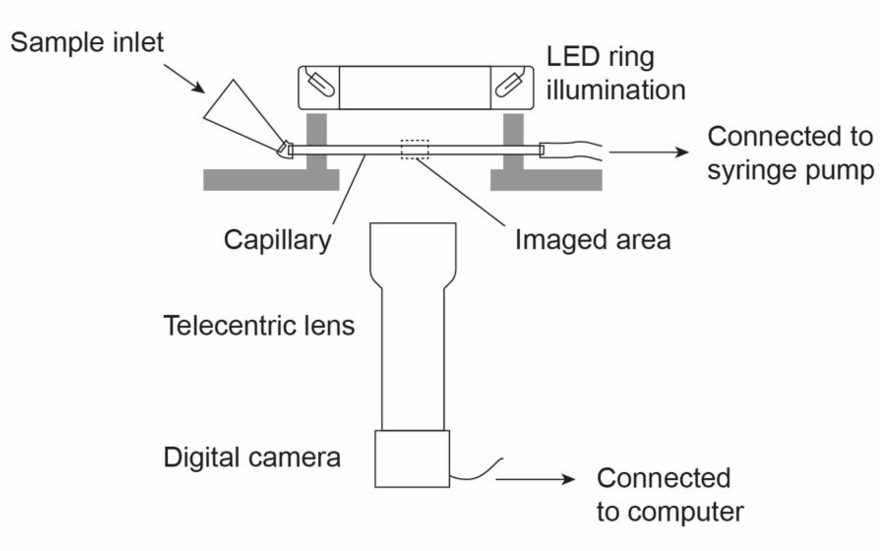


**Supplementary Figure 1.** SMOLT capillary imaging stage. Sample and buffers were added to a sample inlet reservoir and flowed into the capillary using a syringe pump. The capillary was illuminated using a LED ring placed above the capillary to generate dark-field illumination. A 1X telecentric lens and a non-cooled CMOS digital camera were located under the capillary. The digital camera was connected to a computer from which images were acquired.

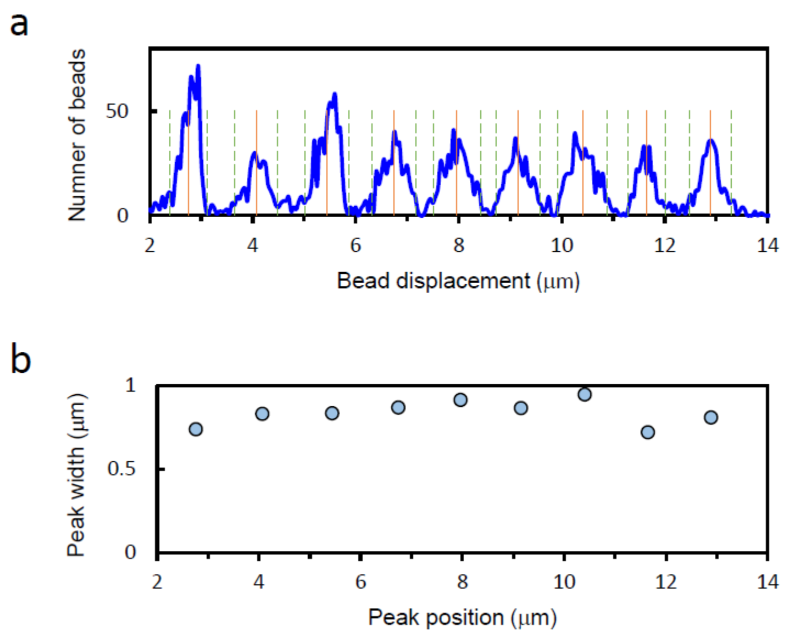


**Supplementary Figure 2.** Precision of bead displacement measurements in SMOLT. (**a**) Bead displacement histogram from a multiplexed experiment where Long-Probes of different length were used (see Fig. 3 in main text). Orange lines show the mean displacement of the beads in the peak. We estimated the precision of bead displacement measurements as the peak width calculated as four times the standard deviation of the displacement of the beads in the peak (distance between green dashed lines). (**b**) Peak width as a function of peak position for each of the peaks shown in **a**.

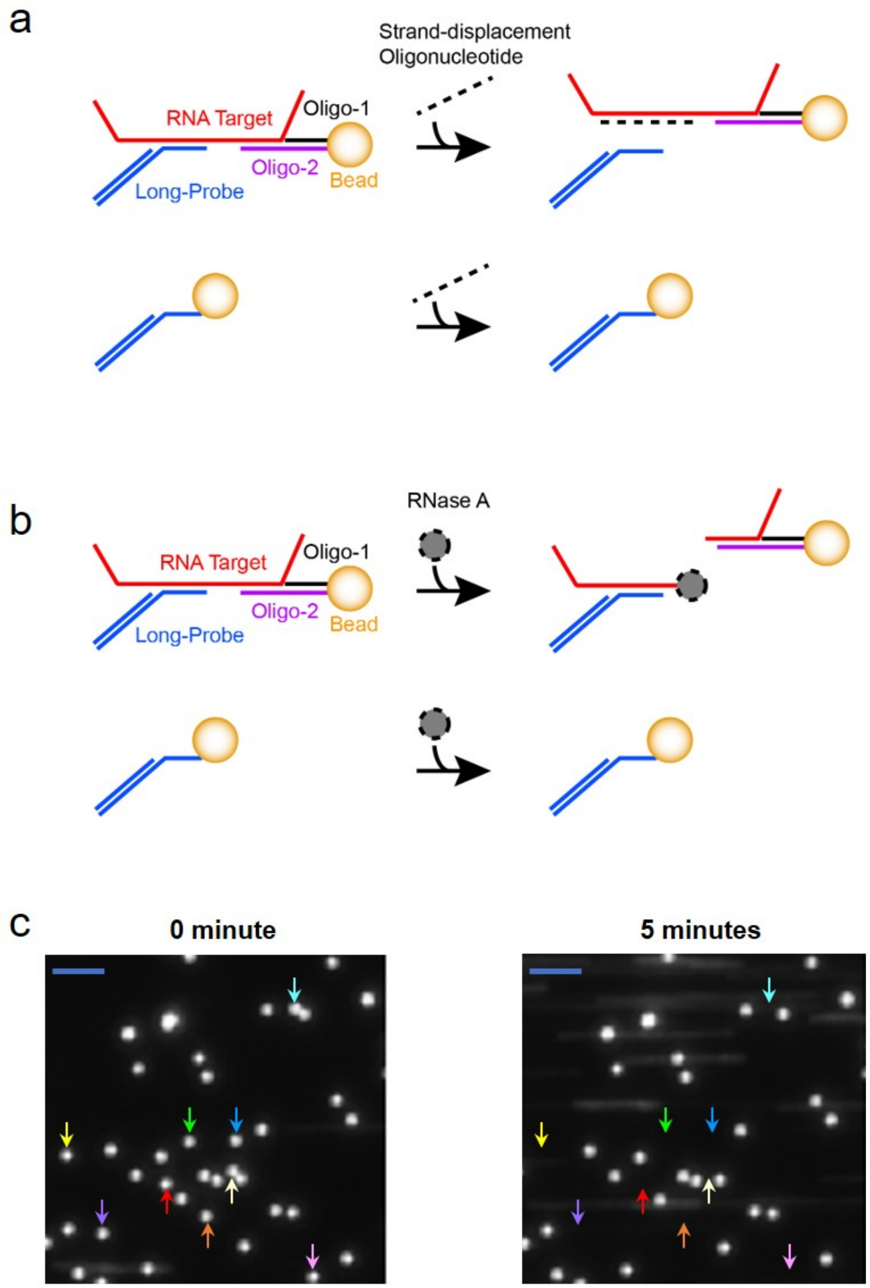


**Supplementary Figure 3.** Disruption step mechanism and disruption experiment images**.** Schematic representation of disruption using (**a**) Strand-displacement DNA oligonucleotides; or (**b**) the RNase A enzyme. (**a, top panel**) Strand-displacement oligonucleotides are designed to be complementary to the rRNA target at the probe binding location plus ≥12 nucleotides up-/down-stream sequences. With such design, the oligonucleotides bind the target and replace the probes (see text). In this manner, beads are disconnected from their Long-Probe tether and removed from the capillary by the flow. (**b, top panel**) RNase A enzyme cleaves the RNA target, resulting in the disconnection of the bead from the Long-Probe tether. (**a-b, bottom panels**) When a bead is non-specifically attached to the Long-Probe, strand-

displacement oligonucleotides or RNase A cannot disconnect the beads from the Long-Probe. Thus, such beads remain after the disruption step. (**c**) Representative capillary images obtained during a disruption experiment. Only a small area from the entire image is shown. In this experiment, *C. glabrata* was spiked into human whole-blood After determining bead displacements, a solution containing strand-displacement oligonucleotide disruptors was flowed continuously into the capillary for 5 minutes. An image was taken before adding disruptors (i.e. 0 minute), and after 5 minutes. A bead is considered disrupted if it is present at the 0-minute image but not present at the 5-minute image. Colored arrows point to examples of disrupted beads; each color corresponds to a different bead. Similar images were obtained in each bead disruption experiment of this study, n> 200. Scale bars, 30 µm.

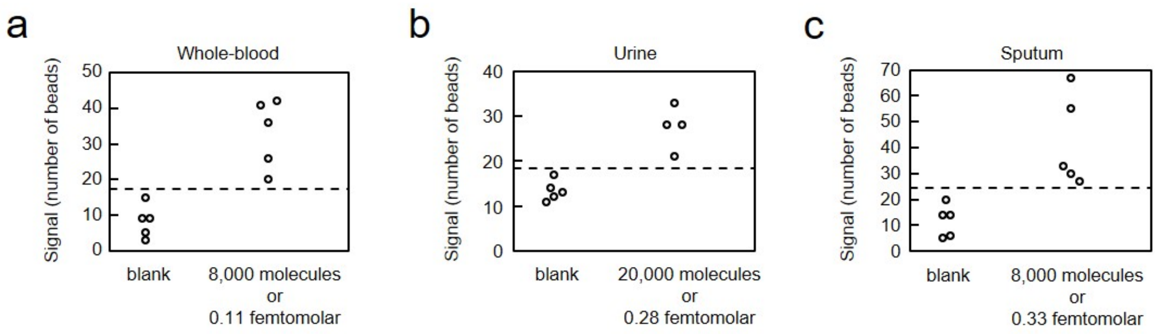


**Supplementary Figure 4.** SMOLT LOD of synthetic RNA oligonucleotides spiked into human **(a)** whole blood, 0.12 mL **(b)** urine, 0.12 mL and **(c)** sputum, 0.04 mL. Independent replicates for non-spiked samples (blank) and LOD concentration are shown. Number of target molecules and target concentration are indicated for each body fluid. The cutoffs (black dashed line) were defined as the mean plus two times the SD of the blank samples signal in the corresponding body fluid. Analysis using unpaired two-tailed *Student's* t-test showed that SMOLT signal from samples with the LOD concentration in each body fluid is significantly different from the signal from corresponding blank samples, each with $p<0.01$. *p*-values are **(a)** $p= 0.00083$, **(b)** $p= 0.00071$, **(c)** $p= 0.0064$.

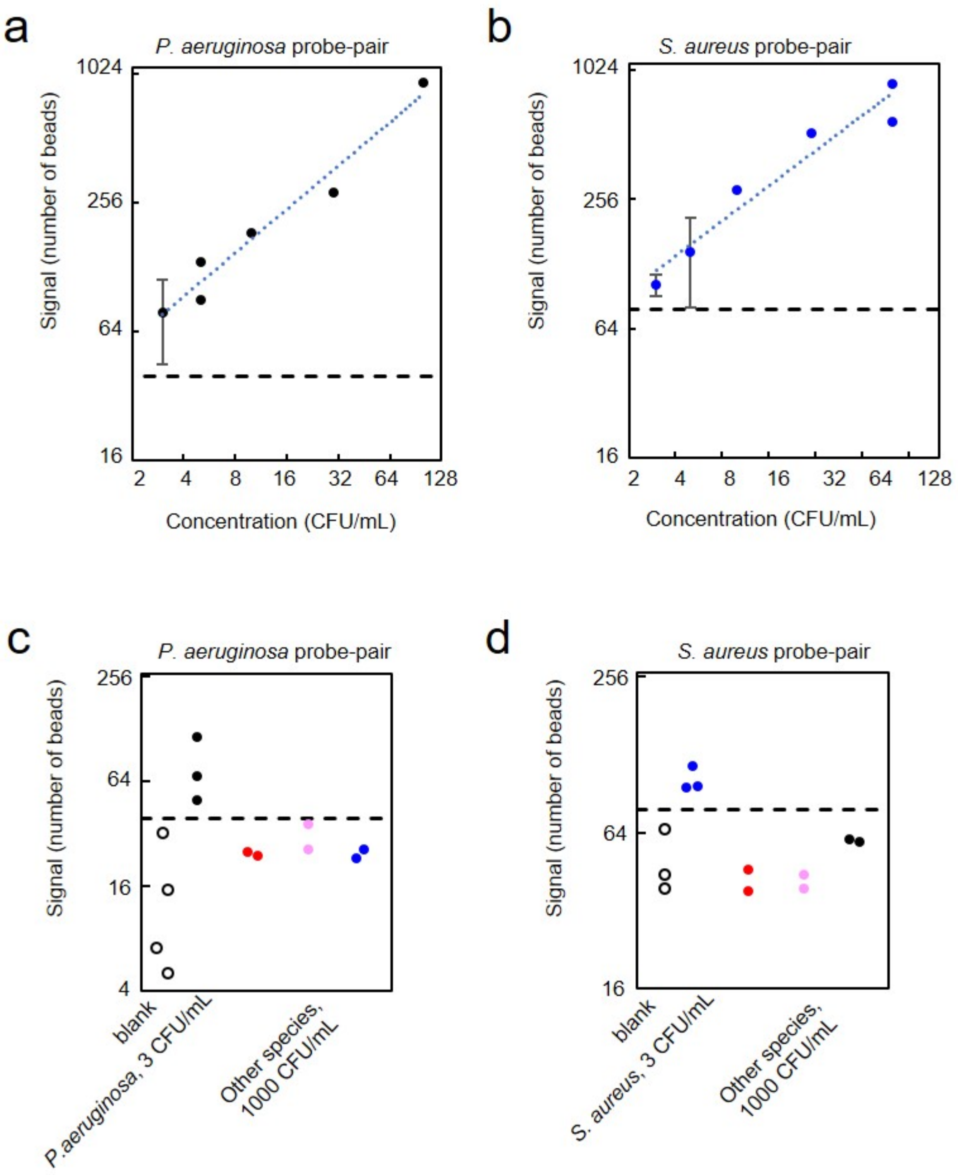


**Supplementary Figure 5.** SMOLT detection of *P. aeruginosa and S. aureus* in human whole blood. Human whole blood samples were spiked with *P. aeruginosa and S. aureus* cells at indicated concentrations. (**a**,**b**) Detection of *P. aeruginosa and S.aureus* across the range of clinical concentrations. Data plotted on Log2 scale for both axes. The signal increased with increasing concentrations. The dotted blue line in each panel is a linear function fitted to the data(**a**) *P. aeruginosa* probe-pair detecting *P. aeruginosa* ($R^2$= 0.0.9619). (**b**) *S. aureus* probe-pair detecting *S. aureus* ($R^2$= 0935). The cutoffs (black dashed line) were defined above the mean plus two times the SD of the blank samples signal for each probe-pair. (**c**,**d**) LOD and cross-reactivity of the (**c**) *P.aeruginosa* probe-pair; (**d**) *S.aureus* probe-pair. Blank samples (empty circles), *K. pneumoniae* (red circles), *E.coli* (pink circles), *P. aeruginosa* (black circles), *S. aureus* (blue circles). The LOD was defined as the concentration for which three measurements were above cutoff with no measurement below cutoff. At each LOD concentration, the signal was significantly different from the blank signal, each with p<0.05.Unpaired two-tailed *t*-test was used for comparing two groups and *p*-values are **(c)** *p= 0.016* for detection of *P.aeruginosa,* **(d)** *p=*

*0.0076* for detection of *S.aureus*. Data **(a,b)** are represented as a single independent experiment or as mean ± SD. Number of experiments for detection of *P. aeruginosa* at concentrations 3 CFU $mL^{-1}$, 5 CFU $mL^{-1}$, 10 CFU $mL^{-1}$, 30 CFU $mL^{-1}$, 100 CFU $mL^{-1}$ were n = 3, 2, 1, 1, 1, respectively. Number of experiments for detection of *S. aureus* at concentrations 3 CFU $mL^{-1}$, 5 CFU $mL^{-1}$, 10 CFU $mL^{-1}$, 30 CFU $mL^{-1}$, 100 CFU $mL^{-1}$ were n = 3, 3, 1, 1, 2, respectively. Source data are provided as a Source Data file.

**Supplementary Table 1.** Background noise reduction based on bead displacement and bead disruption. Table shows the total number of beads detected in the capillary for samples without target (i.e. blank) before background noise reduction and the number of beads counted after imposing the requirement of bead displacement alone and in combination with the requirement of bead disruption.

| Body fluid type | probe-pair | Total number of beads in blank sample | Number of beads that are displaced the same distance that signal beads are displaced | Number of beads that are displaced the same distance that signal beads are displaced AND detach from the capillary during the disruption step |
|---|---|---|---|---|
| blood | Pan-fungal | 10126 | 1405 | 29 |
| | | 12489 | 1178 | 21 |
| | | 7261 | 408 | 14 |
| | | 6356 | 351 | 17 |
| blood | *C. albicans* | 4916 | 141 | 1 |
| | | 7645 | 945 | 0 |
| | | 9586 | 1181 | 0 |
| blood | *C. glabrata* | 8069 | 1711 | 6 |
| | | 7931 | 2250 | 8 |
| | | 10426 | 3075 | 25 |
| | | 8257 | 1383 | 10 |
| blood | Synthetic RNA target | 11213 | 1039 | 11 |
| | | 11962 | 1100 | 17 |
| | | 11759 | 1037 | 12 |
| | | 11323 | 762 | 4 |
| | | 15008 | 790 | 4 |
| urine | | 6885 | 937 | 17 |
| | | 6815 | 1079 | 19 |
| | | 11386 | 347 | 14 |
| | | 9335 | 302 | 15 |
| | | 6292 | 655 | 14 |
| sputum | | 10264 | 1028 | 17 |
| | | 6200 | 676 | 12 |
| | | 6634 | 419 | 9 |
| | | 6441 | 442 | 14 |
| | | 6046 | 396 | 15 |
| | **average** | **8870.19** | **962.96** | **12.50** |

**Supplementary Table 2.** SMOLT probes and strand-displacement DNA oligonucleotides used in this study.

| Probe-pair target | Type of Probe | Probe sequence | Strand-displacement DNA oligonucleotide |
|---|---|---|---|
| *C. albicans* | Probe segment in Long-Probe | CAGCAGCATCCACCAGCAGT | CAAGCGTGTCTACAGCAGCATCCACCAGCAGTCCGTCGTAAAAC |
| | Probe segment in Oligo-2 | TTGATCGTTAAACGTGCCCCGG | ACCAGTTCTAAGTTGATCGTTAAACGTGCCCCGGACGGCCATAAAG |
| *C. glabrata* | Probe segment in Long-Probe | TCCCAAAGTGGTACTCTCCAAATTAC<br>TGCGAGAGTCCCAAGCCCACG | GGCAAAGTACAGTCCCAAAGTGGTACTCTCCAAATTACAACTCGGGCACC *and*<br>CCCCTTGCCTCTCGTGGGCTTGGGACTCTCGCAGCTCACTGGGCC |
| | Probe segment in Oligo-2 | CCTTCCCTTTCAACAATTTCACGTACTT | CTGATCAAATGCCCTTCCCTTTCAACAATTTCACGTACTTTTTCACTCTCTT |
| Pan-fungal | Probe segment in Long-Probe | CTTATTGTGTCTGGACCTGGTGAGTT | CTCAATCTGTCAATCCTTATTGTGTCTGGACCTGGTGAGTTTCCCCGTGTTGAGTC |
| | Probe segment in Oligo-2 | ACTTTGATTTCTCGTAAGGTGCCGAG | CCCAGAACCCAAAGACTTTGATTTCTCGTAAGGTGCCGAGTGCGTCAATAAAAGA |
| Synthetic RNA target used in Fig. 1 | Probe segment in Long-Probe | ACAAGTTTATATTCAGTCATTTTCAGCAGG | Not applicable because RNase A was used for disruption when this probe-pair was used. |
| | Probe segment in Oligo-2 | ATGGCTGCTTCTAAGCCAACATCCTGG | |

**Supplementary Table 3.** Alignments of two fungal rRNA sequences targeted by SMOLT probes in *Candida* detection experiments. One rRNA region is targeted by a *C. albicans*-specific probe. Another is targeted by a pan-fungal probe. Alignment includes representative fungi, bacteria, and human sequences. The rRNA sequences that are targeted by the SMOLT probes are in blue. Flanking sequences outside of the probe-binding region are highlighted in gray. Within the probe-binding region, mismatched nucleotides are shown with red font. Gaps in sequence alignment are show by the dashes ("-"). These multiple sequence alignments were generated using the ClustalW.

| Organism | Species name | rRNA region targeted by a *C. albicans*-specific probe | rRNA region targeted by a pan-fungal probe |
|---|---|---|---|
| Gram-negative bacteria | *Enterobacter cloacae* | GUGGAGCUG---------------AAAUCA------------GUCGA | GCG-----UGGCUUCCGGAGCUAACGCG----UUAAGUCGACCGCCU |
| | *Escherichia coli* | GUGGAGCUG---------------AAAUCA------------GUCGA | GCG-----UGGCUUCCGGAGCUAACGCG----UUAAGUCGACCGCCU |
| | *Klebsiella pneumoniae* | GUGGAGCUG---------------AGACCA------------GUCGA | GCG-----UGGCUUCCGGAGCUAACGCG----UUAAAUCGACCGCCU |
| | *Pseudomonas aeruginosa* | CGUAAGCUC---------------UGGCUG------------GUCGA | AUC-----UUAGUGGCGCAGCUAACGCG----AUAAGUCGACCGCCU |
| Gram-positive bacteria | *Enterococcus faecalis* | -------UU---------------CGGUCA------------GCCGC | CCCU----UCAGUGCUGCAGCAAACGCA----UUAAGCACUCCGCCU |
| | *Enterococcus faecium* | -------UA---------------CGAUCA------------GCCGC | CCCU----UCAGUGCUGCAGCUAACGCA----UUAAGCACUCCGCCU |
| | *Staphylococcus aureus* | -----GCCC---------------AGAAGA------------GCCGC | CCCC----UUAGUGCUGCAGCUAACGCA----UUAAGCACUCCGCCU |
| | *Streptococcus pneumoniae* | -----GCCC---------------AGAAGA------------GCCGC | GGGU----UUAGUGCCGUAGCUAACGCA----UUAAGCACUCCGCCU |
| Target sequence bound by probe | | ------------C-CGGGGCACGUUUAACGAUCAA------------ | ------------CUCGGCACCUUACGAGAAAUCAAAGU--------- |
| Fungi | *Aspergillus fumigatus* | -------GCCGUC-CGGCGGGCGUUAACGACCAACUUAGAACUGGU | UAUGAUGACCCGCUCGGCACCUUACGAGAAAUCAAAGUUUUUGGGUU |
| | *Candida albicans* | -------GCCGUC-CGGGGCACGUUUAACGAUCAACUUAGAACUGGU | UUUAUUGACGCAAUCGGCACCUUACGAGAAAUCAAAGUCUUUGGGUU |
| | *Candida glabrata* | --------CCGUCGCUUGCUGCGAUUAACGAUCAACUUAGAACUGGU | UUUAGUGACCCACUCGGCACCUUACGAGAAAUCAAAGUCUUUGGGUU |
| | *Candida krusei* | --------UUUUC-AGUCCCGCGGUUAACAACCGACUUAGAACUGGU | UUUG----CCCACUCGGCACCUUACGAGAAAUCAAAGUUUUUGGGUU |
| | *Candida parapsilosis* | --------UCGUC-CGGAUCACGCUUAACGAUCAACUUAGAACUGGU | UUUAUUGACGCAAUCGGCACCUUACGAGAAAUCAAAGUCUUUGGGUU |
| | *Cryptococcus neoformans* | -------GGCGUC-CGGCGUACGCUUAACAACCAACUUAGAACUGGU | UCUC-UGACUGGGUCGGCACCUUACGAGAAAUCAAAGUCUUUGGGUU |
| | *Cryptococcus gattii* | -------GGCGUC-CGGCGUACGCUUAACAACCAACUUAGAACUGGU | UCUC-UGACUGGGUCGGCACCUUACGAGAAAUCAAAGUCUUUGGGUU |
| | *Fusarium solani* | -------GCCGUC-CGGCGUGCGGUUAACAACCAACUUAGAACUGGU | UAUUUUGACUCGUUCGGCACCUUACGAGAAAUCAAAGUGCUUGGGCU |
| Human | *Homo sapiens* | GCGGCGCCUCGCCUCGGCCGGCGCCUAGCAGCCGACUUAGAACUGGU | UCCCAUGACCCGCCGGGCAGCUUCCGGGAAACCAAAGUCUUUGGGUU |
