# Supplementary material for: Ultra-sensitive and rapid detection of nucleic acids and microorganisms in body fluids using single-molecule tethering
Source: Nat Commun. 2020 Sep 22;11:4774. doi: 10.1038/s41467-020-18574-7 (PMC7508858; doi:10.1038/s41467-020-18574-7)
Supplement: Supplementary file 2 — Reporting Summary [file 41467_2020_18574_MOESM2_ESM.pdf]

nature research

Corresponding author(s): Alfredo Celedon

Last updated by author(s): Aug 12, 2020

Reporting Summary

Nature Research wishes to improve the reproducibility of the work that we publish. This form provides structure for consistency and transparency in reporting. For further information on Nature Research policies, see Authors & Referees and the Editorial Policy Checklist .

Statistics

For all statistical analyses, confirm that the following items are present in the figure legend, table legend, main text, or Methods section.

n/a	Confirmed	
☐	☒	The exact sample size (*n*) for each experimental group/condition, given as a discrete number and unit of measurement
☐	☒	A statement on whether measurements were taken from distinct samples or whether the same sample was measured repeatedly
☐	☐	The statistical test(s) used AND whether they are one- or two-sided<br>*Only common tests should be described solely by name; describe more complex techniques in the Methods section.*
☐	☒	A description of all covariates tested
☐	☒	A description of any assumptions or corrections, such as tests of normality and adjustment for multiple comparisons
☐	☒	A full description of the statistical parameters including central tendency (e.g. means) or other basic estimates (e.g. regression coefficient) AND variation (e.g. standard deviation) or associated estimates of uncertainty (e.g. confidence intervals)
☐	☒	For null hypothesis testing, the test statistic (e.g. *F*, *t*, *r*) with confidence intervals, effect sizes, degrees of freedom and *P* value noted<br>*Give P values as exact values whenever suitable.*
☒	☐	For Bayesian analysis, information on the choice of priors and Markov chain Monte Carlo settings
☒	☐	For hierarchical and complex designs, identification of the appropriate level for tests and full reporting of outcomes
☒	☐	Estimates of effect sizes (e.g. Cohen's *d*, Pearson's *r*), indicating how they were calculated

Our web collection on statistics for biologists contains articles on many of the points above.

Software and code

Policy information about availability of computer code

Data collection	Software Octave (version 5.1.0) was used to acquire images and to analyze them.
Data analysis	Data analysis was conducted in Microsoft Excel (version 16.0.13001.20266).

For manuscripts utilizing custom algorithms or software that are central to the research but not yet described in published literature, software must be made available to editors/reviewers. We strongly encourage code deposition in a community repository (e.g. GitHub). See the Nature Research guidelines for submitting code & software for further information.

Data

Policy information about availability of data

All manuscripts must include a data availability statement. This statement should provide the following information, where applicable:

- Accession codes, unique identifiers, or web links for publicly available datasets
- A list of figures that have associated raw data
- A description of any restrictions on data availability

The authors declare that the data supporting the findings of this study are available within the paper and its supplementary information files.
Source data for Figures 1f-h, 2a-c and Supplementary Fig. 5a, b available in the supplementary material.

Field-specific reporting

Please select the one below that is the best fit for your research. If you are not sure, read the appropriate sections before making your selection.

Life sciences study design

All studies must disclose on these points even when the disclosure is negative.

Sample size	Sample size between 3-6 were used throughout this study in Oligonucleotide and Fungi detection experiments. Within each experimental group, each human body fluid sample was independently spiked with an oligonucleotide, microorganism or none (for blank samples). Some concentration titration experiments had smaller sample size (1-2) in bacteria detection experiments. Sample size was not predetermined by a statistical method.
Data exclusions	No data was excluded
Replication	All the results have been obtained independently at least twice. The data was generated by four operators using ten instruments, at least two samples for each body fluid, and several organisms. For most of the experimental conditions, 3 to 6 repeats were obtained.
Randomization	Samples/Organisms were randomly assigned to different instruments and operators.
Blinding	Experiments were not blinded. Operators spiked samples with different oligonucleotide/organisms concentrations and collected the results of the assay. Experimental results were not adjusted in any way and subjective observation or judgment was not used to add or exclude data.

Reporting for specific materials, systems and methods

We require information from authors about some types of materials, experimental systems and methods used in many studies. Here, indicate whether each material, system or method listed is relevant to your study. If you are not sure if a list item applies to your research, read the appropriate section before selecting a response.

Materials & experimental systems

n/a	Involved in the study
☒	☐ Antibodies
☒	☐ Eukaryotic cell lines
☒	☐ Palaeontology
☒	☐ Animals and other organisms
☐	☒ Human research participants
☒	☐ Clinical data

Methods

n/a	Involved in the study
☒	☐ ChIP-seq
☒	☐ Flow cytometry
☒	☐ MRI-based neuroimaging

Human research participants

Policy information about studies involving human research participants

Population characteristics	a scientist conducting the experiments provided urine samples with informed consent.
Recruitment	scientist self-provided samples
Ethics oversight	*Identify the organization(s) that approved the study protocol.*

Note that full information on the approval of the study protocol must also be provided in the manuscript.
